# Cost-effectiveness of interventions to improve hand hygiene in healthcare workers in middle-income hospital settings: a model-based analysis

**DOI:** 10.1016/j.jhin.2018.05.007

**Published:** 2018-10

**Authors:** N. Luangasanatip, M. Hongsuwan, Y. Lubell, D. Limmathurotsakul, P. Srisamang, N.P.J. Day, N. Graves, B.S. Cooper

**Affiliations:** a Mahidol–Oxford Tropical Medicine Research Unit, Faculty of Tropical Medicine, Mahidol University, Bangkok, Thailand; b School of Public Health, Queensland University of Technology, Brisbane, Australia; c Centre for Tropical Medicine and Global Health, Nuffield Department of Clinical Medicine, University of Oxford, Oxford, UK; d Department of Tropical Hygiene, Faculty of Tropical Medicine, Mahidol University, Bangkok, Thailand; e Department of Pediatrics, Sanpasithiprasong Hospital, Ubon Ratchatani, Thailand; f Institute of Health and Biomedical Innovation, Queensland University of Technology, Brisbane, Australia

**Keywords:** Cost-effectiveness, Hand hygiene, Healthcare workers, Bloodstream infections, *Staphylococcus aureus*, Hospital

## Abstract

**Background:**

Multi-modal interventions are effective in increasing hand hygiene (HH) compliance among healthcare workers, but it is not known whether such interventions are cost-effective outside high-income countries.

**Aim:**

To evaluate the cost-effectiveness of multi-modal hospital interventions to improve HH compliance in a middle-income country.

**Methods:**

Using a conservative approach, a model was developed to determine whether reductions in meticillin-resistant *Staphylococcus aureus* bloodstream infections (MRSA-BSIs) alone would make HH interventions cost-effective in intensive care units (ICUs). Transmission dynamic and decision analytic models were combined to determine the expected impact of HH interventions on MRSA-BSI incidence and evaluate their cost-effectiveness. A series of sensitivity analyses and hypothetical scenarios making different assumptions about transmissibility were explored to generalize the findings.

**Findings:**

Interventions increasing HH compliance from a 10% baseline to ≥20% are likely to be cost-effective solely through reduced MRSA-BSI. Increasing compliance from 10% to 40% was estimated to cost US$2515 per 10,000 bed-days with 3.8 quality-adjusted life-years (QALYs) gained in a paediatric ICU (PICU) and US$1743 per 10,000 bed-days with 3.7 QALYs gained in an adult ICU. If baseline compliance is not >20%, the intervention is always cost-effective even with only a 10% compliance improvement.

**Conclusion:**

Effective multi-modal HH interventions are likely to be cost-effective due to preventing MRSA-BSI alone in ICU settings in middle-income countries where baseline compliance is typically low. Where compliance is higher, the cost-effectiveness of interventions to improve it further will depend on the impact on hospital-acquired infections other than MRSA-BSI.

## Introduction

Hospital-acquired infections (HAIs) are a major cause of morbidity and mortality among hospitalized patients [1]. HAIs are also associated with a substantial economic burden due to longer hospital stays and additional antibiotic costs [2]. The risk of infection in developing countries is two to 20 times higher than in developed countries [3]. In Thailand, among hospitalized patients, the point prevalence of nosocomial infection has been estimated at 6.5% and ∼250,000 patients are believed to have an HAI each year [4].

Direct patient contact with healthcare workers (HCWs) transiently contaminated with nosocomial pathogens is believed to be the primary route of transmission. Improving HCW hand hygiene compliance can minimize the impact of this transmission route and reduce the incidence of nosocomial infection [5]. A multi-modal intervention including system change, training and education, observation and feedback, reminders, and a hospital safety climate has been developed and promoted by the World Health Organization (WHO). This campaign (referred to as WHO-5) has been shown to be effective in increasing hand hygiene compliance [5], [6]. Hand hygiene promotion is also relatively easy to implement and requires a modest level of investment. Nevertheless, in many healthcare settings, particularly in low- and middle-income countries, compliance remains poor and reports of rates of <10% may be typical [7], [8], [9].

Transmission dynamic models are useful tools to help understand the likely impact of interventions to control communicable diseases. Moreover, their use in health-economic evaluations of interventions that reduce transmission is essential to fully capture intervention benefits. However, whereas several studies have used dynamic models to consider hospital infections [10], [11], economic evaluations of hand hygiene interventions have used only static models and have largely neglected developing countries where the need for appropriate investment is greatest [12], [13], [14], [15], [16]. Whereas one previous cohort study in Vietnam concluded that a hand hygiene intervention was cost-saving (i.e. the reduction in costs from HAIs averted exceeded the intervention cost), there have been no systematic attempts to quantify the levels of investment in hand hygiene promotion under which it remains cost-effective or to explore how appropriate levels of investment depend on pre-intervention levels of hand hygiene compliance [16].

The aims of this study are to develop a dynamic model-based framework for evaluating the cost-effectiveness of hand hygiene promotion interventions and use it to evaluate the cost-effectiveness of such interventions in a middle-income country. Our analysis is informed by data from a typical regional hospital in Thailand, a middle-income country with a gross domestic product (GDP) per capita approximately equal to the world median. We focus on MRSA-BSI as this is one of the most serious and best-studied types of infection in ICU patients, there is clear evidence of frequent patient-to-patient transmission of MRSA, and evidence that such transmission can be interrupted by improved hand hygiene [17], [18], [19]. Hand hygiene interventions should also reduce other types of MRSA infections and infections with other organisms. However, since these are harder to quantify, we take a conservative approach by focusing on MRSA-BSI alone and are therefore likely to underestimate the true health benefits of the intervention [20].

## Methods

### Overall description

Transmission dynamic and decision analytic models were combined to simulate the transmission dynamics and evaluate the impact and cost-effectiveness of hand hygiene interventions. Two ICU settings were considered: a paediatric intensive care unit (PICU) and an adult intensive care unit (adult ICU). Epidemiological and economic parameters were derived from detailed local data from a typical tertiary hospital in North-east Thailand. Information about catchment area, staff:patient ratios, and further details about the ICUs have been described elsewhere [21], [22]. Incidence of hospital-acquired and healthcare-associated BSI from this and other hospitals in the same region have also been reported in previous studies [22], [23].

### Transmission dynamic model

**Figure 1:**
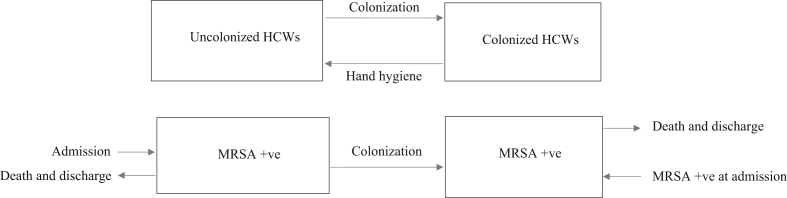
Model structure.

A previously described deterministic host–vector model was constructed to simulate MRSA transmission dynamics in an ICU (Figure 1) [24]. Patients can be admitted to the ICU either uncolonized or colonized with MRSA. Uncolonized patients can become colonized or infected by contact with transiently colonized HCWs. HCWs can be decolonized by performing hand hygiene. Colonized patients have a specified risk of developing MRSA-BSI. The model outputs the number of newly colonized patients, the number of MRSA-BSIs and the number of deaths over one year under different levels of hand hygiene compliance. The model was implemented in R, using the package ‘deSolve’ to numerically solve the equations [25], [26]. Model outcomes were fed into the decision analytic model. Full technical details are given in Appendix A.

Model parameters were obtained from various sources (Table I). MRSA carriage data were derived from a previous observational study in North-east Thailand [22], [27]. Estimates of the per-contact transmission probabilities (HCW to patient and patient to HCW) were derived using these data, combined with previous estimates of the probability of transmission from a colonized patient to HCW (see Appendix A) [27], [40]. The rates at which colonized patients acquired an MRSA-BSI were estimated from the average number of cases per year at each ward divided by the expected number of colonized bed-days (estimated from the carriage data). Risk of death due to MRSA-BSI was taken from an observation study in the same setting [22]. The number of beds, number of HCWs per shift, rates of ICU discharge, ward-specific contact rates, and the baseline hand hygiene compliance were directly observed from the same hospital.

**Table I:** Model parameters

Parameters		Paediatric ICU			Adult ICU			Distribution	Source
		Mean	2.5^th^ percentile	97.5^th^ percentile	Mean	2.5^th^ percentile	97.5^th^ percentile		
Transmission dynamic model	Proportion of admissions colonized with MRSA	0.063	0.029	0.108	0.087	0.038	0.139	Beta	[23]
	HCW–patient transmission probability per contact	0.0065	0.0028	0.0105	0.0113	0.0061	0.0192	Beta	[23], [24]
	Patient–HCW transmission probability per contact	0.132	0.078	0.194	0.132	0.078	0.194	Beta	[24]
	Patient/HCW contacts per day (per patient)	8	–	–	8	–	–		Direct observation
	HCW/patient contacts per day (per HCW)	14	–	–	9	–	–		Direct observation
	Infection rate from colonized (per day)	0.0013	0.0007	0.0021	0.0013	0.0008	0.0020	Gamma	Database
	Probability of attributable death given MRSA-BSI	0.439	0.338	0.5390	0.439	0.338	0.539	Beta	[27]
	Removal rate of uncolonized patient (1/LOS) (per day)	0.164	–	–	0.173	–	–		Database
	Removal rate of colonized patients (1/LOS) (per day)	0.164	–	–	0.173	–	–		Database
	No. of beds	7	–	–	10	–	–		Direct observation
	No. of HCWs (per shift)	4	–	–	9	–	–		Direct observation
	Hand hygiene compliance (baseline)	0.1	–	–	0.1	–	–		Direct observation
Economic model	Cost (US$, 2016)								
	Hand hygiene intervention (per ward per year)	675.4	281.4	1069.4	719.9	305.0	1134.6	Gamma	[28], [29], [30]
	ICU bed day	47.3	15.3	71.8	47.3	15.3	71.8	Gamma	Database, [32]
	General ward bed day	5.5	2.1	10.5	5.5	2.1	10.5	Gamma	Database, [32]
	Treatment MRSA-BSI (per case)	142.8	265.4	478.6	214.2	95.6	398.1	Gamma	[33], [35], [36]
	Excess length of stay due to MRSA-BSI (per case)	2.2	–0.1	4.6	1.4	–1.3	4.1	Normal	Database
	Utility post-ICU	0.72	0.56	0.88	0.72	0.56	0.88	Beta	[36], [37], [38], [39]
	QALYs gained per death averted (3% discounted)	17.95	10.48	24.67	10.31	7.92	12.76	Gamma	Database, [21]

ICU, intensive care unit; MRSA, meticillin-resistant *Staphylococcus aureus*; HCW, healthcare worker; BSI, bloodstream infection; LOS, length of hospital stay; QALY, quality-adjusted life-year.

### Economic evaluation

Cost-utility analysis was performed from a healthcare provider's perspective. The cost of the hand hygiene intervention was estimated over a period of one year. Health benefits were measured with a lifetime horizon and a 3% discounting rate. Costs were adjusted to 2016 values with the exchange rate of 33 Thai Baht per US$1 [41]. This economic evaluation is in accordance with the Consolidated Health Economic Evaluation Reporting Standards (CHEERS) statement [42].

There were two main cost components: cost of hand hygiene promotion; and costs associated with MRSA-BSI. The latter includes costs of additional hospital stay and antibiotic treatment (Table I). The cost of the hand hygiene campaign accounted for staff time and materials used. As there is limited information for the cost of hand hygiene interventions in Thailand, we derived this information from a survey conducted in Australia from the national hand hygiene campaign implementing interventions similar to WHO-5 assuming the same time per bed-day requirements but applying Thai pay-scale salaries for registered nurses with two and 10 years' experience [28], [29]. Costs of alcohol hand rub (AHR) were included in the model; other material costs were assumed to be negligible. The intervention was assumed to increase AHR use 3.5-fold (range: 2–5) [30]. Baseline AHR use was directly observed in all local paediatric and adult ICUs. We found similar amounts of AHR used in both types of ICUs, therefore the average AHR use (98 L per ICU at 10% compliance) was applied in both wards. Associated costs assumed a market price of US$2.4 per litre provided by the national pharmaceutical supplier in Thailand [31]. Total hand hygiene intervention costs were estimated to be US$675 and US$720 per ward per year in the PICU and adult ICU, respectively, due to the difference in number of beds per ward (seven and 10 beds, respectively).

Costs associated with MRSA-BSI were estimated from additional stay and treatment. Hospitalization cost was calculated as the excess length of stay due to MRSA-BSI multiplied by the cost per bed-day. Retrospective data from routine clinical and microbiological laboratory databases at the local hospital (2003–2010) were used to identify MRSA-BSI cases. Additional stay due to infection was estimated with a multi-state model accounting for time-dependent bias using the ‘etm’ package within R [43], [44]. The economic value of a bed-day should reflect the opportunity cost of an occupied bed, which can be quantified by asking healthcare providers for their willingness to pay (WTP) for an unoccupied bed-day [45]. This opportunity cost is typically much lower than the cost calculated with an accounting approach (hospital budget divided by the total patient bed-days) [32]. In the absence of WTP per ICU bed-day in Thailand, the accounting cost was estimated using local hospital financial data and this was multiplied by the ratio of bed-day costs estimated with WTP and accounting approaches reported in a previous study [32].

Treatment for MRSA-BSI was assumed to require a 14-day course of vancomycin with dose regimens following treatment guidelines for hospital-acquired MRSA-BSI. Drug costs were obtained from the Drug Medical Supply Information Center [33].

Estimates of life expectancy among post-ICU patients were taken from a previous study in North-east Thailand [21]. Health-related quality of life among patients after ICU discharge who had MRSA-BSI during the ICU stay was assumed to be the same as general post-ICU patients. These utility values were taken from the literature [36], [37], [38], [39]. The median utility of 0.72 was used in the base case with a range from 0.56 to 0.88 and assumed to be constant.

### Analyses

Four scenarios with different baseline versus post-intervention hand hygiene compliance values were considered: (a) 10% vs 20%; (b) 10% vs 40%; (c) 10% vs 60%; and (d) 40% vs 60%. These are consistent with results from a systematic review where odds ratios were estimated to be 6.5 and 11.8 for WHO-5 and WHO-5 plus other interventions among studies using an interrupted time-series design (with a baseline compliance of 10%, these would give post-intervention compliance values of 42% and 57%, respectively) [46].

In each comparison, point estimates of incremental costs (ΔC) and QALYs gained (ΔQ) due to the intervention and the incremental cost-effectiveness ratio (ICER; ΔC/ΔQ) were calculated. The threshold willingness to pay per QALY gained (λ) was taken as GDP per capita (US$4848), and a threshold value of three times GDP per capita was considered in a scenario analysis [47]. The latter threshold corresponds to WHO criteria for a cost-effective intervention and the former to a highly cost-effective intervention [34]. Interventions with ICERs below the chosen WTP threshold are, by definition, cost-effective. Probabilistic sensitivity analyses (PSA) were undertaken using 10,000 Monte Carlo iterations where parameters were sampled from specified distributions (Table II). Simulation results were used to calculate the monetary net benefit (MNB), which is defined as λ*Q – C for each level of achieved hand hygiene compliance and the distribution of incremental monetary net benefits (IMNB) for each comparison (λ*ΔQ – ΔC). In addition, the maximum level of investment in the intervention at which it would still be cost-effective was calculated as monetary incremental benefits (λ*ΔQ) plus the saving in treatment costs from averted infections.

**Table II:** Economic evaluation of hand hygiene promotion in paediatric and adult ICUs (2016)

HHC	MRSA-BSI avoided	Deaths averted per 10,000 bed-days	Incremental cost^a^ (US$)	QALYs gained	ICER	Average monetary net benefits^b^ (95% CI) (US$)			Average IMNB^b^ (95% CI) (US$)		
Paediatric ICU											
Baseline (HHC 10%)						30,355,764	19,974,308	43,152,328	–	–	–
HHC 20%	0.093	0.1593	653.34	0.69	951.00	30,358,438	19,983,010	43,153,618	2674	153	7694
HHC 40%	0.1318	0.2258	644.05	0.97	660.84	30,359,838	19,986,042	43,154,482	4074	555	11,054
HHC 60%	0.143	0.2453	641.32	1.06	605.59	30,360,250	19,986,829	43,154,760	4486	680	12,024
HHC 40% vs HHC 60%	0.011	0.0196	672.81	0.08	7959.42	30,360,250	19,986,829	43,154,760	(263)	(818)	411
Adult ICU											
Baseline (HHC 10%)						21,563,698	16,822,741	26,943,092	–	–	–
HHC 20%	0.2326	0.2796	660.46	0.96	684.77	21,567,718	16,828,312	26,945,105	4020	926	9213
HHC 40%	0.3243	0.3898	636.25	1.35	470.60	21,569,619	16,829,969	26,946,027	5921	1622	13,187
HHC 60%	0.3503	0.4211	629.30	1.46	430.14	21,570,164	16,830,431	26,946,285	6466	1822	14,288
HHC 40% vs HHC 60%	0.0260	0.0313	713.93	0.11	6431.80	21,570,164	16,830,431	26,946,285	(176)	(772)	536

ICU, intensive care unit; HHC, hand hygiene compliance; MRSA-BSI, meticillin-resistant *Staphylococcus aureus* bloodstream infection; QALY, quality-adjusted life year; ICER, incremental cost-effectiveness ratio; IMNB, incremental monetary net benefit.

a Per ward per year.

b Monetary net benefits reported per ward (total admission) assuming a willingness to pay for a QALY of US$4840 (160,000 Thai baht, exchange rate; US$1 = 33 Thai baht).

A series of hypothetical scenarios with different assumptions about the transmissibility and prevalence of MRSA colonization at admission were considered. The ward reproduction number (*R*_A_), the expected number of MRSA cross-transmissions resulting from a single colonized patient during a single ward stay, assuming all other patients on the ward are susceptible, was varied between 0.5 and 5, while prevalence of MRSA colonization on ICU admission was varied between 1% and 15%. Changes in costs and health outcomes under different baseline compliance and improvement levels were calculated and combined to evaluate the cost-effectiveness of such interventions in terms of the IMNB. The maximum level of investment at which the intervention would still be cost-effective, the prevalence reduction, and final prevalence in all scenarios were also determined.

## Results

**Figure 2:**
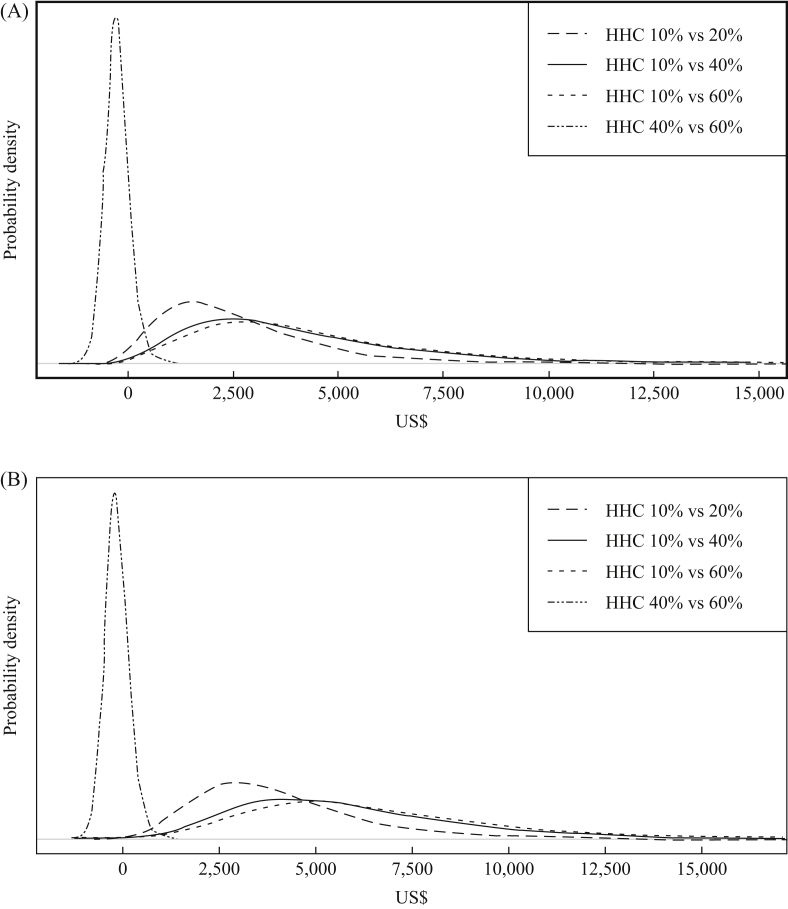
Base case and scenario analyses plotting probability density of incremental net monetary benefits for hand hygiene intervention across four different hand hygiene compliance (HHC) scenarios (baseline compliance at 10% compared with post intervention at 20%, 40% and 60% and baseline compliance at 40% compared with post intervention at 60%) at willingness to pay per quality-adjusted life-year gained of US$4848 for paediatric intensive care unit (A) and adult intensive care unit (B).

Under base case assumptions (with a pre-intervention hand hygiene compliance of 10%), a multi-modal hand intervention (WHO-5) is highly likely to be cost-effective in both PICU and adult ICU settings if it increases hand hygiene compliance to ≥20% (Table II and Appendix B). Conversely, if baseline compliance is 40%, the expected IMNB is likely to be negative, indicating that the intervention is unlikely to be cost-effective solely as a result of reducing MRSA-BSIs (Figure 2).

Risk of death due to MRSA-BSI in our study hospital was estimated to be between two- and three-fold higher than in high-income countries [22], [35]. However, a scenario analysis showed that the intervention is still highly cost-effective if a lower mortality risk estimated from high-income settings is used instead (Table III). When the WTP threshold was three times GDP per capita (US$14,545), under base case assumptions (with a pre-intervention hand hygiene compliance of 10% and post-intervention compliance of 40%), the IMNBs were positive for both PICU and adult ICU.

**Table III:** Scenario analysis for base case (baseline hand hygiene compliance of 10% vs 40% hand hygiene compliance)

Setting	Incremental outcomes		ICER	Mean IMNB^a^^b^ (95% CI) (US$)	Mean (95% CI) maximum investment^a^^b^ (US$)
	Costs (US$)	QALYs^a^			
Paediatric ICU, per ward, per year (2016)					
Base case	644	0.97	661	4074 (555–11,054)	4839 (1344–11,668)
Cost of hand hygiene intervention (5-fold increase from US$ 675 to US$3375)	3369	0.97	3457	1453 (–2919–9586)	4833 (1320–12,306)
QALY gained per death averted amongst post-ICU patients (lower bound = 10.48 instead of 17.95)	644	0.58	1113	2156 (32–6262)	2836 (776–6880)
No utility weights (LE = 24.93 instead of 17.95)	644	1.36	474	5953 (1127–15,458)	6634 (1814–16,069)
Low attributable mortality due to MRSA-BSI (at 20%)	644	0.45	1422	1533 (–156–4850)	2213 (588–5488)
High attributable mortality due to MRSA-BSI (at 50%)	644	1.13	571	4815 (840–12,742)	5498 (1547–13,345)
Include additional stay in general wards given BSI (12.8 days)	636	0.97	652	4161 (642–11,118)	4837 (1350–11,725)
Adult ICU, per ward, per year (2016)					
Base case	636	1.35	471	4020 (926–9213)	5513 (1560–13,224)
Cost of intervention (5-fold increase from US$720 to US$3600)	3535	1.35	2623	3102 (–1722–10,312)	6723 (2498–13,735)
QALY gained per death averted among post-ICU patients (lower bound = 7.92 instead of 10.31)	636	1.05	606	4460 (1178–9822)	5184 (1927–10,572)
No utility weights (LE = 14.32 instead of 10.31)	636	1.90	335	8580 (2709–18,321)	9304 (3455–19,002)
Low attributable mortality due to MRSA-BSI (at 20%)	636	0.89	712	3696 (788–8661)	4421 (1542–9432)
High attributable mortality due to MRSA-BSI (at 50%)	636	1.56	409	6905 (2132–14,717)	7632 (2884–15,403)
Include additional stay in general wards given BSI (12.8 days)	620	1.35	460	6017 (1743–12,935)	6743 (2518–13,648)

a QALY, quality-adjusted life year; ICER, incremental cost-effectiveness ratio; IMNB, incremental monetary net benefit; CI, confidence interval; ICU, intensive care unit; LE, life expectancy; MRSA-BSI, meticillin-resistant *Staphylococcus aureus* bloodstream infection; HHC, hand hygiene compliance.

b Incremental monetary net benefits and maximum investment at which the intervention would still be cost-effective assuming a willingness to pay for a QALY of $US4,840 (160,000) exchange rate; $US 1 = 33 Thai baht).

**Figure 3:**
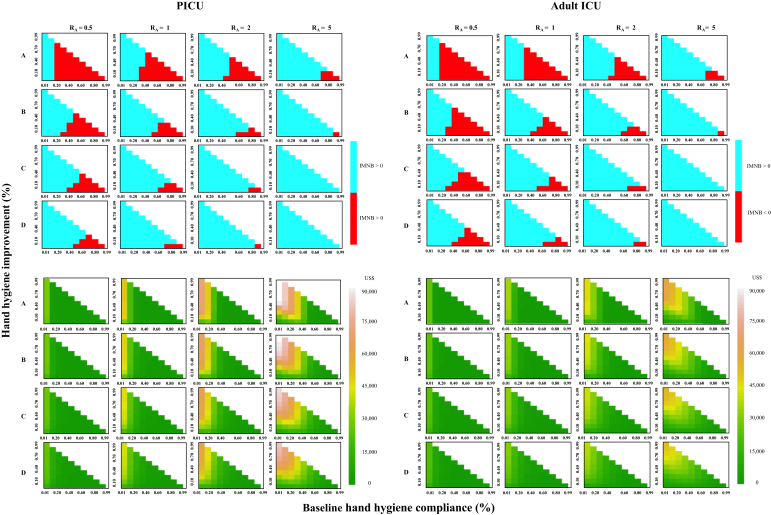
Incremental monetary net benefit (IMNB) (top); blue for IMNB >0 (cost-effective) and red for IMNB <0 (not cost-effective) and maximum intervention cost at which the intervention would still be cost-effective (bottom) from hypothetical scenario analyses with different values of baseline hand hygiene compliance, compliance improvement, and the ward reproduction number (R_A_) for paediatric intensive care unit (PICU) (left) and adult ICU (right) at willingness to pay per quality-adjusted life-year gained of US$4848. Proportion of admissions colonized with meticillin-resistant *Staphylococcus aureus*: (A) 0.01, (B) 0.05, (C) 0.10, (D) 0.15.

**Figure 4:**
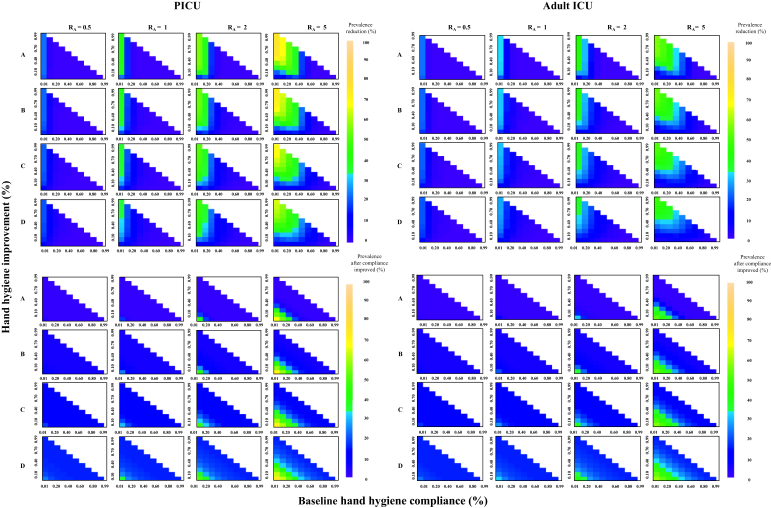
Prevalence reduction of meticillin-resistant *Staphylococcus aureus* (MRSA) carriage due to intervention (top) and equilibrium prevalence of MRSA carriage after improved hand hygiene compliance (bottom) from hypothetical scenario analyses with different values between baseline hand hygiene compliance, compliance improvement and the ward reproduction number (R_A_) for paediatric intensive care unit (PICU) (left) and adult ICU (right). Proportion of admissions colonized with meticillin-resistant *Staphylococcus aureus*: (A) 0.01, (B) 0.05, (C) 0.10, (D) 0.15.

In hypothetical scenario analyses, the hand hygiene intervention was found to be cost-effective in most scenarios, especially when there was high transmissibility and a high prevalence of MRSA colonization on admission (Figure 3). In the situations where the transmissibility is low (R_A_ = 0.5), where prevalence of MRSA colonization at admission is 5%, and where baseline compliance is ≤20% in both the PICU and the adult ICU, the intervention is always cost-effective even with only a 10% compliance improvement using the cost estimates in Table I. When the baseline compliance is ≤20%, the intervention will always be cost-effective if the intervention cost per year is less than US$1557 in the PICU and US$888 in the adult ICU, provided that the intervention increases compliance by ≥10%. Prevalence reduction of MRSA carriage and the final prevalence of all hypothetical scenarios are shown in Figure 4.

## Discussion

Hand hygiene promotion using the WHO multi-modal campaign is likely to be highly cost-effective for ICU settings in Thailand where baseline compliance is low (≤20%) solely as a result of preventing MRSA-BSI. Factors that tended to make the intervention more cost-effective were low baseline compliance, high prevalence of colonization at admission, and high rates of transmission. With higher baseline compliance, the intervention may often still be highly cost-effective as a result of reduced MRSA-BSI alone if rates of MRSA carriage on ICU admission or ICU transmission are sufficiently high.

Because we ignored impacts of the intervention on other types of HAI (other MRSA infections and infections with other pathogens) our analysis is conservative and likely to underestimate health benefits. MRSA-BSIs represent only 5.1% of hospital-acquired BSIs in North-east Thailand, whereas Gram-negatives account for 67.6% [23]. Evidence linking increased hygiene with reduced infection rates is less compelling for Gram-negative organisms than it is for MRSA, but there are credible reports that such an association exists [48], [49]. Some of the strongest evidence concerns multidrug-resistant *Acinetobacter* spp., where a segmented regression analysis found that a hand hygiene intervention was associated with a substantial change in incidence of infections with extensively drug-resistant *Acinetobacter* spp. in Taiwan [13]. *Acinetobacter* spp. has also been reported to be a frequent contaminant of the hands of HCWs in endemic settings in South-east Asia, strengthening the evidence for a causal link between increased hand hygiene and reduced infections [50]. Since *Acinetobacter* spp. are the largest single cause of hospital-acquired BSI in North-east Thailand, if infections with these organisms can be reduced substantially by improved hand hygiene, the implications for our analysis could be substantial [22]. Whereas our work provides an analytical framework for such an evaluation, better data on the epidemiology of *Acinetobacter* spp. and the effects of hand hygiene are needed to inform it.

To the best of our knowledge, our study is the first economic evaluation of a hand hygiene intervention to make use of a dynamic model in a developing country [12], [13], [14], [15], [16]. In previous economic evaluations in high-income countries, Pittet *et al.* (in Switzerland), and Chen *et al.* (in Taiwan) used data from observational studies to estimate reductions in infections due to hand hygiene interventions. Pittet *et al.* concluded that if only 1% of the observed reduction was due to the intervention it would have been cost-saving. Chen *et al.* also concluded that their intervention was likely to be cost-saving. Huis *et al.* (in the Netherlands) used trial data to inform a cost-effectiveness analysis, assuming a linear relationship between hand hygiene compliance and reduced infections, concluding that the intervention was likely to be cost-effective if the willingness to pay for a 1% reduction in the HAI rate was about US$6000. A study in the UK also concluded that hand hygiene interventions were likely to be cost-saving even if the reduction in rates of HAI were as low as 0.1%. As in our study, this report explicitly calculated QALY gains. However, unlike the other studies, staff time was not accounted for when costing the intervention. A previous study in a middle-income country (Vietnam) concluded that a hospital-wide hand hygiene promotion was cost-saving [16].

Direct comparison of these findings with ours is difficult for three reasons. First, only one of the previous studies quantified benefits in terms of final health outcomes (QALYs) [12]. Second, bed-day costs are much greater in high-income settings and account for most of the costs associated with HAIs. In developing country settings, costs of antibiotics to treat infections are likely to be the dominant cost [16]. Third, there are important differences in aims and methodology. We focused on MRSA-BSI (where we have strong evidence that it can be reduced by hand hygiene), reasoning that if the intervention is cost-effective for this outcome alone then it should certainly be cost-effective overall. We also made use of important methodological advances, accounting for the expected non-linear association between hand hygiene compliance and infection rates using a mathematical model, avoiding time-dependent biases when estimating increased length of stay, valuing bed-days based on opportunity cost rather than using an accounting approach, and estimating life-years gained by preventing mortality using data from a large linked-database study [10], [21], [24], [43], [32]. These advances, combined with the much lower bed-day costs, help explain why we estimated the cost per infection to be a few hundred dollars, whereas studies in other countries in both high- and middle-income countries estimated it to be a few thousand [13], [14], [15], [16].

Our study has some limitations, the most important of which is that data are not yet available that allow us to include other pathogens in the model. In addition, since local information on the resources used for the hand hygiene intervention is limited, we were dependent on estimates of resource used for hand hygiene interventions from studies outside Thailand. A further limitation is that we evaluated the intervention over only a one-year post-intervention period where the initial investment would be most. Additional costs to maintain the compliance in the later years are difficult to quantify but likely to be less costly than the first year.

In conclusion, effective multi-modal hand hygiene interventions are likely to be cost-effective in ICU settings in typical middle-income countries due to reduced incidence of MRSA-BSI alone under a wide range of circumstances. When this is not so, the cost-effectiveness of interventions to further improve hand hygiene will depend on the impact on other infections and other pathogens. Further work is needed to quantify this.

## Footnotes

Appendix A Supplementary data related to this article can be found at https://doi.org/10.1016/j.jhin.2018.05.007
