# Supplementary material for: Cost-effectiveness of interventions to improve hand hygiene in healthcare workers in middle-income hospital settings: a model-based analysis
Source: J Hosp Infect. 2018 Oct;100(2):165–75. doi: 10.1016/j.jhin.2018.05.007 (PMC6204657; doi:10.1016/j.jhin.2018.05.007)

**Appendix A. Model description and parameter estimation**

*List of parameters and definition*

| Parameters | Definition |
| --- | --- |
| $U_{pat}$ | Number of MRSA (–ve) patients on ward at one time |
| $C_{pat}$ | Number of MRSA (+ve) patients on ward at one time |
| $n_{pat}$ | Number of beds (U_pat+C_pat) |
| $U_{HCW}$ | Number of MRSA (–ve) HCWs per shift |
| $C_{HCW}$ | Number of MRSA (+ve) HCWs per shift |
| $n_{HCW}$ | Number of total HCWs per shift (U_HCW+C_HCW) |
|  |  |
| $\mu$ | Removal rate of uncolonized patient (1/mean length of stay) (per day) |
| $\gamma$ | Removal rate of colonized patients (1/mean length of stay) (per day) |
| $\pi$ | Proportion of admission with colonized |
| *c* | Patient/HCW contact per day (per patient) |
| $P_{HP}$ | Transmission probability from HCW to patient per contact |
| *c′* | HCW/patient contact per day (per HCW) |
| $P_{PH}$ | Transmission probability from patient to HCW per contact |
| $HHC$ | Hand hygiene compliance |
| $\lambda$ | Hand hygiene rate defined as$\frac{HHC*c'*n_{pat}}{\left( 1-HHC \right)*n_{HCW}}$ |

*Model equations*

$\frac{{dU}_{pat}}{dt}= - \mu*U_{pat}-c*P_{HP}*U_{pat}*\frac{C_{HCW}}{n_{HCW}}+\left( 1-\pi\right)*(\mu*U_{pat}+\Upsilon*C_{pat})$ (1)

$\frac{{dC}_{pat}}{dt}= - \Upsilon*C_{pat}+c*P_{HP}*U_{pat}*\frac{C_{HCW}}{n_{HCW}}+ \pi*(\mu*U_{pat}+ \Upsilon*C_{pat})$ (2)

$\frac{{dU}_{HCW}}{dt}= -\left( c^{'}*P_{PH}*C_{pat}*\frac{U_{HCW}}{n_{HCW}} \right)+ \lambda*C_{HCW}$ (3)

$\frac{{dC}_{HCW}}{dt}= \left( c^{'}*P_{PH}*C_{pat}*\frac{U_{HCW}}{n_{HCW}} \right)- \lambda*C_{HCW}$ *(4)*

*Estimates of transition probability from HCWs to patient (*$P_{HP}$*) and the ward reproduction number (R_A_)*

From Worby *et al*. [1] we have an estimate from our data of the force of infection,$\beta$, arising from a single colonized patient.

Assuming the number of colonized HCWs can be approximated by the quasi-equilibrium values, we can use equations (1)–(4) to derive the expression for$P_{HP}$ in terms of $\beta$ and other parameters.

$P_{HP}=\frac{\beta*\left( c^{'}*P_{PH}+ \lambda*n\_HCW \right)}{c*c'*P_{PH}}$

We used directly observed values of $c, c^{'}, n_{HCW}, n_{pat}, and HHC,$ whereas $P_{PH}$ was adopted from elsewhere, estimated by McBryde *et al.* [2].

The ward reproduction number (R_A_), the number of secondary cases arising from one primary case in a completely susceptible population, is given by

$R_{A}= \frac{{c*P_{HP}*c}^{'}*P_{PH}*(n_{pat} - 1)}{\gamma*(\lambda*n_{HCW} +P_{PH}*c^{'})}$

*References*

[1] Worby C. Statistical inference and modelling for nosocomial infections and the incorporation of whole genome sequence data. PhD thesis. UK: University of Nottingham; 2012.

[2] McBryde ES, Bradley LC, Whitby M, McElwain DL. An investigation of contact transmission of methicillin-resistant *Staphylococcus aureus*. J Hosp Infect 2004;58:104–8.

**Appendix B**

Results from probabilistic sensitivity analysis (PSA) showing 10,000 iterations for each of the four different hand hygiene compliance (HHC) scenarios (baseline compliance at 10% compared with post intervention at 20%, 40% and 60% and baseline compliance at 40% compared with post intervention at 60%) at willingness to pay per quality-adjusted life-year (QALY) gained of US$4,848 for paediatric intensive care unit (PICU) (upper) and adult ICU (lower).


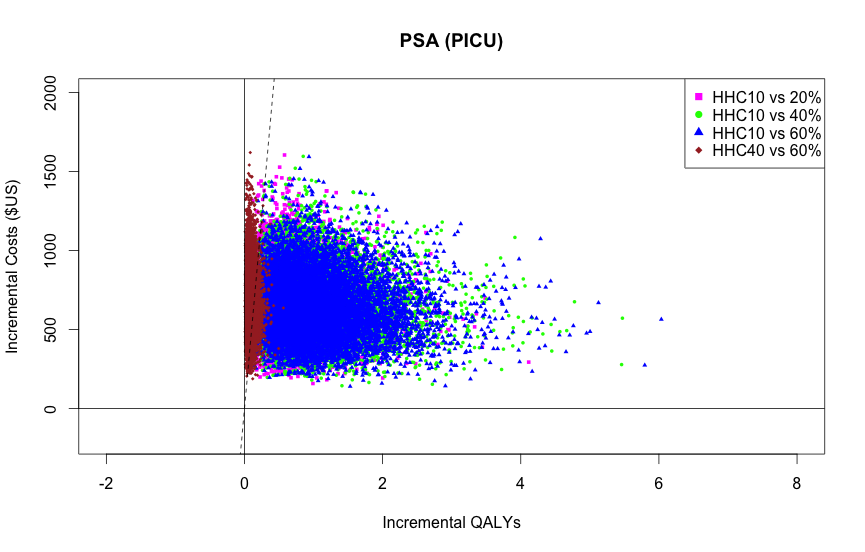


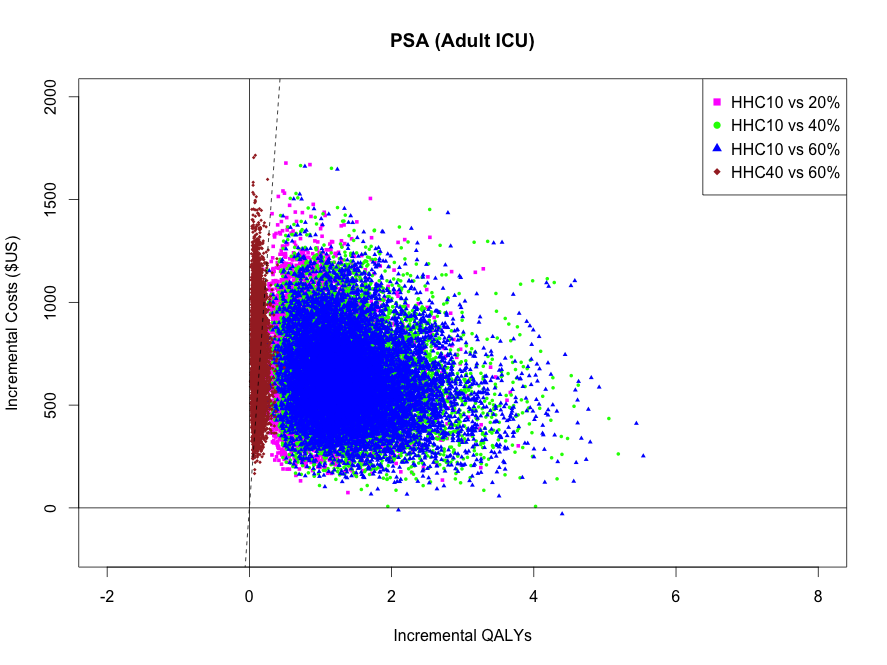

Supplement: Multimedia component 1 [file mmc1.docx]
